# Uncertainty about others’ trustworthiness increases during adolescence and guides social information sampling

**DOI:** 10.1038/s41598-022-09477-2

**Published:** 2022-05-10

**Authors:** I. Ma, B. Westhoff, A. C. K. van Duijvenvoorde

**Affiliations:** 1 Department of Psychology, New York University, New York, USA; 2 Institute of Psychology, Leiden University, Leiden, The Netherlands; 3 Leiden Institute for Brain and Cognition, Leiden, The Netherlands

**Keywords:** Psychology, Human behaviour

## Abstract

Adolescence is a key life phase for developing well-adjusted social behaviour. An essential component of well-adjusted social behaviour is the ability to update our beliefs about the trustworthiness of others based on gathered information. Here, we examined how adolescents (*n* = 157, 10–24 years) sequentially sampled information about the trustworthiness of peers and how they used this information to update their beliefs about others’ trustworthiness. Our Bayesian computational modelling approach revealed an adolescence-emergent increase in uncertainty of prior beliefs about others’ trustworthiness. As a consequence, early to mid-adolescents (ages 10–16) gradually relied less on their prior beliefs and more on the gathered evidence when deciding to sample more information, and when deciding to trust. We propose that these age-related differences could be adaptive to the rapidly changing social environment of early and mid-adolescents. Together, these findings contribute to the understanding of adolescent social development by revealing adolescent-emergent flexibility in prior beliefs about others that drives adolescents’ information sampling and trust decisions.

## Introduction

Adolescence is a life-phase accompanied by a strong social reorientation^1^. Adolescents spend more time with peers^2–4^, are susceptible to peer influence^5,6^, and aim to achieve and maintain a positive peer status^1,7–10^. Violations of trust, such as social rejection, gossiping, and other negative peer interactions are exceptionally detrimental to adolescents’ mental health and social development^7,11^. It is therefore imperative for adolescents to sample information about the trustworthiness of their peers to update their beliefs and adapt their behaviour accordingly. For example, information can be sampled by asking close friends for their opinion about a specific peer or by observing how they treat others. When the sampled information indicates that the peer violates the trust of others, the adolescent should update their belief about that peer’s trustworthiness (e.g., from likely trustworthy to likely untrustworthy) and adapt their behaviour towards that peer accordingly.

However, sparse samples might not be representative of the peer’s true trustworthiness. An untrustworthy peer might sometimes act in a trustworthy manner. Insufficient information can therefore result in erroneously trusting an untrustworthy peer or not trusting a trustworthy peer. Not much is known about the age differences in trustworthiness information sampling, despite of the relevance of this process to social development during adolescence. Understanding how adolescents determine the quantity of their trustworthiness information samples, sheds light on the adaptive changes that underlie social development and may expose potential improvements.

Given the social reorientation during adolescence, it might be intuitive to expect that adolescents focus more on peers and therefore excessively sample information about peers compared to children or adults. However, a recent study using a novel task and computational model, we identified three distinct factors that underlie the process of information sampling about others’ trustworthiness in adults^12^ and the findings give rise to more nuanced hypotheses about information sampling in adolescents. The main factors that were identified in the study were: (1) prior beliefs about trustworthiness, (2) uncertainty about the prior belief, and (3) uncertainty tolerance. The first factor, the *prior beliefs about trustworthiness*, reflects an individual’s initial expectation about others’ trustworthiness before any information is sampled. Past studies in adults show that a biased prior belief subsequently biases how information is sampled and how the belief is updated in the light of new information^13,14^. For example, adults were more likely to update their belief about another person if the novel information was consistent with their prior belief^14^, and actively sample information to support their prior belief^15^. Prior beliefs about trustworthiness might show age differences across adolescence. Empirical studies have shown that initially placed trust increases from childhood to adulthood^16–19^, suggesting a potential shift in prior beliefs about trustworthiness during adolescence (but see^20,21^). One of the aims of the current study is therefore to assess if prior beliefs about trustworthiness indeed shift during adolescence and affect information sampling and/or bias decisions to trust or not trust a peer.

**Figure 1:**
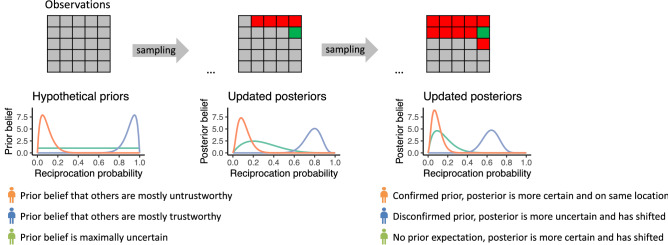
Illustration of how prior belief distributions update to posterior beliefs. The grid represents sampled information about trustworthiness. A green tile indicates that the sample resulted in an observation of trustworthiness, red tiles represent observations of untrustworthiness, and grey tiles are not sampled. At the start all tiles are grey as no samples have been drawn yet and therefore the current belief distribution about trustworthiness is the prior belief distribution. The belief distributions update with each sample. The orange, green and blue lines in the plots represent three hypothetical subjects’ prior belief distributions and their corresponding, updated posterior belief distribution. The updated posteriors belief distributions in the middle reflect an intermediate belief stage when there are 4 red and 1 green tile. The updated posterior belief distributions on the right reflect the scenario where even more samples were drawn (10 red and 1 green tile). These three hypothetical subjects were selected to illustrate that the posterior beliefs can be quite different depending on the prior expectation (mean) and the prior uncertainty (variance) of the prior belief distribution. The orange prior distribution reflects the expectation that lower reciprocation probabilities are more likely. The observed outcomes match that prior expectation. The posterior uncertainty therefore decreases but the posterior mean does not update. The blue prior distribution reflects a belief that higher reciprocation probabilities are more likely. The sample outcomes disconfirm this belief and the posterior belief therefore shows a large update (a shift in the mean) and becomes more uncertain (higher variance). The green prior distribution has a maximal uncertainty, i.e., a belief that all reciprocation probabilities are equally likely. The posterior belief shows both a large update and reduced uncertainty.

The second important factor when sampling information about others is the *uncertainty about prior beliefs*^12^. This reflects the variation an individual expects in the trustworthiness of others. We first explain this concept in more detail before discussing potential changes during adolescence. For example, an individual with high uncertainty about their prior belief expects more variation in trustworthiness between different trustees. In contrast, an individual with low uncertainty about their prior belief expects that there will be little variation in trustworthiness between different trustees. There can be uncertainty about any prior belief; one individual might expect that all trustees are untrustworthy (low uncertainty), another could expect that everyone is trustworthy (low uncertainty), and yet another might expect that some are trustworthy while others are not (high uncertainty). Each new sample updates both the belief and the uncertainty. The updated result is a posterior belief and uncertainty about the posterior belief, respectively. Adults were shown to sample information until their posterior uncertainty dropped below a level to which they were tolerant to uncertainty^12^. Uncertainty about prior (and posterior) beliefs thereby influence the quantity of information samples, such that higher uncertainty likely results in more sampling (Fig. 1)^12^.

Little is known about the development of uncertainty about prior beliefs during adolescence, possibly because beliefs and especially uncertainty are difficult to observe directly in choice behaviour and often require assessment through Bayesian computational models. Uncertainty about prior beliefs of trustworthiness is likely to change during adolescence, as changes take place in the set and frequency of social behaviours displayed by peers during early adolescence (e.g., courtship or competitive behaviour such as gossiping). Transitioning from primary to high school may alter the adolescents’ expectations about new peer groups and new group dynamics. Normatively, cues that signal novelty in the social environment should indeed increase uncertainty about the generalizability of previously learned social behaviours^22,23^ (e.g., “childish” games such as playing tag may not be socially accepted anymore in high school). Specifically, uncertainty about prior beliefs should *increase* when the environment becomes more volatile, which leads to heightened sensitivity to new information, thereby allowing the individual to update their beliefs more with each new information sample^24^. Given the numerous changes in adolescents’ social lives, we therefore expect an age-related *increase* in their uncertainty about their prior beliefs of peers’ trustworthiness.

The third and final factor is *uncertainty tolerance*, which reflects the level of posterior uncertainty that an individual finds tolerable^12^. As mentioned earlier, this affects the sample quantity together with the uncertainty about prior beliefs, as adults sample until their posterior uncertainty drops below their uncertainty tolerance level^12^. Previous developmental studies showed individual and age-related differences in how tolerant adolescents are to uncertainty by using questionnaires^25,26^ and experimental risky choice tasks that vary the level of outcome uncertainty^25^. In general, these studies using experimental tasks suggest that adolescents are more tolerant to uncertainty, which led them to explore risky gambles more often compared to adults^27,28^ (but see^29,30^). One previous study explored how uncertainty tolerance related to sampling information for monetary rewards. Findings showed that adolescents sample less information about lottery outcomes than children and adults, also suggesting that adolescents are more uncertainty tolerant than children and adults^31^. Whereas these previous studies suggest that uncertainty tolerance might be a trait that underlies risky choice, little is known about how an individual’s level of uncertainty tolerance drives behaviour in the social domain and how this may change during adolescence. Given that information about peers is highly important for adolescents to successfully navigate their changing social environment, adolescents’ uncertainty tolerance in non-social lottery tasks might not generalize to sampling information about peers and instead adolescents might become more uncertainty intolerant with age, especially from early to mid-adolescence.

**Figure 2:**
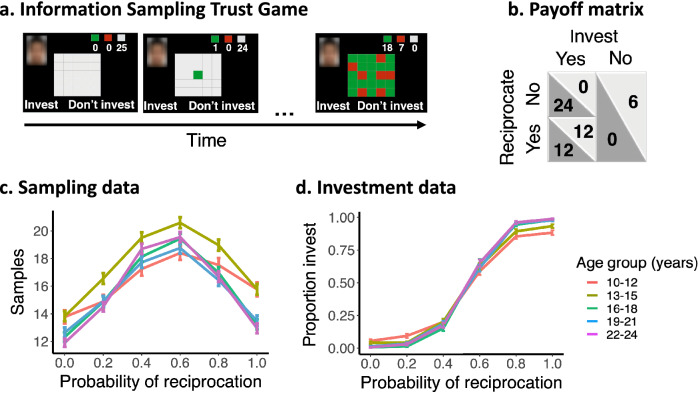
Information Sampling Trust Game and data. (**a**) Task trial sequence example and payoff matrix. On each trial there are 2 players: the investor and a trustee. The participants played in the investor role and could sample a trustee’s reciprocation history with other investors up to 25 times by turning tiles in a 5 by 5 grid. Green = reciprocated trust, red = betrayed trust, grey = not sampled. Investment outcomes were not shown during the task. Six reciprocation probability conditions (*r* = 0.0, 0.2, 0.4, 0.6, 0.8, 1.0) generated the outcomes in the grid. It was clarified in the instructions that they were playing with someone their own age, that the location of the tile was not informative, that each trial would be played with a new unknown trustee, and that the ratio green to red tiles may thus vary between trials. (**b**) Payoff matrix. Participants were told that if they invested, the trustee received the 6 tokens, which would be multiplied by 4 (24 tokens) and subsequently the trustee would decide to either reciprocate by splitting the 24 tokens 50–50, or defect and keep all 24 tokens to themselves. Participants also had the option of not investing by keeping the initial endowment. (**c**) The number of samples (mean and standard error of the mean (s.e.m.)) as a function of reciprocation probability per age group (years). Age groups were created for visualization purposes only and analyses were conducted with age as continuous measure. (**d**) Proportion of investments as function of the generative reciprocation probability for each age group.

In summary, here we examined age differences in 1. prior beliefs about trustworthiness, 2. uncertainty of prior beliefs about trustworthiness, and 3. uncertainty tolerance as factors that potentially may affect age-related differences in information sampling about others’ trustworthiness. Participants (10–24 years, *n* = 157, 75 of which were boys) completed the Information Sampling Trust Game (ISTG, see Fig. 2a). The Trust Game mimics characteristic consequences of trust, such that trusting is beneficial to all involved partners if reciprocated, but trust can also be betrayed. The ISTG extends this paradigm by allowing the participant to sample information about the trustee’s history of trustworthiness before making a decision to trust or not trust.

At the beginning of each trial, participants were endowed with 6 tokens which they could invest (entrust) in the trustee in a single-shot Trust Game (see Fig. 2b for payoff matrix). Participants were told that these trustees previously played this game with 25 different investors in a different experiment and that their decisions to reciprocate or defect were stored in a covered 5 × 5 grid. Participants were given the opportunity to first sample information about the trustee’s reciprocation history before deciding to either invest or not invest. Unbeknownst to participants, the grid outcomes were computer-generated and drawn from the following probabilities: 0.0, 0.2, 0.4, 0.6, 0.8, and 1.0, where 0.0 is completely untrustworthy (all red) and 1.0 is fully trustworthy (all green). Each subject sampled information about 60 different trustees (one trustee per trial). There were no explicit sampling costs other than the time and effort involved in turning tiles. The outcomes of participants’ trust decisions (invest or not invest) after sampling were not shown during the task to avoid changing meta-beliefs about the reliability of the acquired information. Instead, participants were told that 3 trials would be randomly selected at the end of the task and their average amount of tokens would be converted to money and paid to the participant (supplement).

## Results

### Behavioural analyses

On average participants sampled 16.229 (*SD* = 7.532) out of the 25 tiles presented on each trial. We expected based on non-social sampling studies^32^ that participants would sample more when the sample outcomes were less consistently green or red (i.e., when the outcome uncertainty was highest) and we examined the interaction with age. To this end, we used a Robust linear mixed effects model (‘robustlmm’ in R^33^) to predict the number of samples from outcome uncertainty (i.e., the variance in the Bernoulli distribution *r*(1 − *r*) where *r* is the true, experimentally set probability of reciprocation), linear and nonlinear (quadratic) age effects and the age × outcome uncertainty interactions. Full model specification is reported in the Supplement.

We found that participants sampled significantly more when the outcome uncertainty was higher, i.e., when the probability of reciprocation was closer to 0.5 (*B* = 2.138, *P* < 0.001, see Fig. 2c). This effect interacted with the linear (*B* = 0.355, *P* < 0.001) and quadratic effect of age (*B* = − 0.140, *P* = 0.003). There was no significant main effect of age (age linear *P* = 0.065; age quadratic *P* = 0.617). We conducted post-hoc Tukey corrected analyses to further examine this interaction effect with age. A comparison between age groups (10–12; 13–15; 16–18; 19–21; 22–24 years) showed that the effect of outcome uncertainty was significantly less strong in early-adolescents (10–12) compared to older ages (see Supplement for R code and all pairwise comparisons). To examine this further and test whether the number of samples was also affected by trustworthiness level, we next ran an additional mixed effects model in which we explored the effect of age and reciprocation probability on the number of samples. This revealed a significant interaction between reciprocation probability and the linear effect of age (*B* = − 0.17, *P* < 0.001). To follow up on this interaction, we performed a post-hoc test comparing the effect of age between different reciprocation probability bins. This showed that with age, participants sampled less when the trustee was highly trustworthy (reciprocation probability of 0.8: *B* = − 1.130, *P* = 0.021, reciprocation probability of 1.0: *B* = − 1.564, *P* = 0.001) and fully untrustworthy (reciprocation probability of 0.0: *B* = − 1.05, *P* = 0.031), see Fig. 2c and see Supplement for all results and R code.

To examine the invest decisions, we used a generalised linear mixed-effects model (glmer function; lme4 package^34^). We tested whether the invest decisions (i.e., decisions to trust or not) were predicted by trustworthiness (i.e., the reciprocation probability), age (linear and quadratic) and their interactions (see Supplement for full model specification). As expected, we found a significant main effect of trustworthiness (odds ratio (OR) = 30.04, 95% CI[25.92–34.81], *P* < 0.001), showing that the likelihood of investing increased when the trustee was more trustworthy. There was a significant interaction between trustworthiness and the linear effect of age (OR = 2.06, 95% CI[1.80–2.36], *P* < 0.001) and the quadratic effect of age (OR = 0.76, 95% CI[0.67–0.86], *P* < 0.001). There was no significant main effect of age (age linear OR = 1.00, 95% CI[0.86–1.16], *P* = 0.987; age quadratic OR = 1.04, 95% CI[0.90–1.21], *P* = 0.592). We conducted post-hoc Tukey corrected analyses to further examine this interaction effect comparing the age effect within each reciprocation probability. This showed that with age, participants were more likely to invest in highly trustworthy trustees (reciprocation probability 0.8: OR = 1.93, *P* < 0.001; reciprocation probability 1.0 OR = 2.88, *P* < 0.001) and more likely not to invest in highly untrustworthy trustees (reciprocation probability 0.0 OR = 0.37, *P* < 0.001; see Supplement for all pairwise comparisons).

Taken together, we found that early-adolescents were less sensitive to outcome uncertainty. Specifically, even though younger adolescents sampled more information about trustworthy peers than older participants (see Fig. 2c,d), younger participants were less likely to trust peers who were trustworthy and to some extent more likely to trust highly untrustworthy trustees compared to older adolescents. This behaviour also resulted in younger adolescents earning less on this task than older adolescents (see Supplement for analyses on expected reward).

### Computational processes underlying trustworthiness information sampling

Information sampling and age differences therein were well captured by a Bayesian model of information sampling, called the Uncertainty model^12^. At its core, this model has a Bayesian belief distribution over the trustworthiness of the trustee. This belief distribution encompasses the prior belief and the uncertainty about the prior belief. With each sample this belief distribution is updated, resulting in the posterior belief distribution. The probability of stopping information sampling increases as the updated uncertainty drops below the uncertainty tolerance level (see Methods, “Computational model” for the formal description of the Uncertainty model).

We compared the Uncertainty model against three alternative computational models to test if trustworthiness information sampling strategies differed with age (see Supplement for formal descriptions of alternative models). The Sample Cost model and the Threshold model are alternative models developed in a previous study on the ISTG^12^ and the Count model was added to test if a simpler heuristic strategy would be more prevalent in late childhood than at older ages. The *Sample Cost model* uses the Bayesian belief distribution to compute the normative solution for every state. The *Threshold model* is similar to the Uncertainty model but instead of using a Bayesian belief distribution, it is based on the concept of sampling until the ratio between red and green tiles meets a subjective threshold. Finally, the *Count model* is the simplest model and tests if participants are insensitive to the gathered evidence and instead sample a fixed number of tiles. All models allow for decision noise.

**Figure 3:**
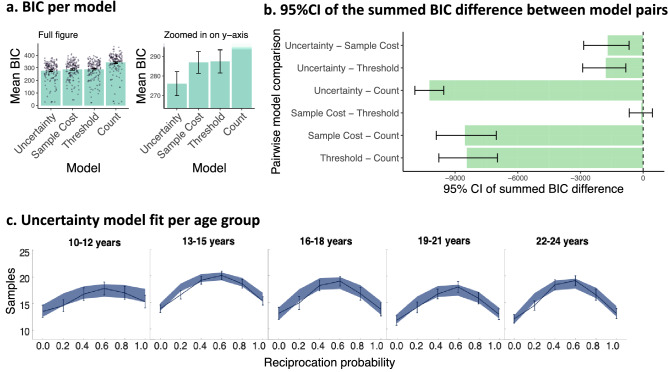
Model fit results. (**a**) The Bayesian Information Criterion (BIC) per model, averaged over participants (bars depict the mean and error bars show the s.e.m). Dots show individual BIC scores. Lower BIC values indicate a better fit, thus showing that the Uncertainty model fits best. BIC scores were computed for each participant and each model. The left panel is the full figure, while the panel on the right is zoomed in as the count model fitted much less well than the other models. (**b**) 95% confidence intervals (CI) of the summed BIC difference between models. Zero indicates no difference between models. Negative values are in favour of the model before the subtraction sign, as lower BIC indicates a better fit. The Uncertainty model fits significantly better than the Sample Cost and Threshold models (95% CI does not contain zero). The BIC scores of a model pair were subtracted from each other for each participant, thereby obtaining one difference score per participant for each model pair. To assess significance, the 95% confidence interval of the BIC difference was computed using bootstrapping with 10^5^ iterations. (**c**) Alignment between the uncertainty model predictions and behavioural data across age. Age is grouped for visualization purposes. The shaded area is the s.e.m. of the data that was simulated with the participants’ estimated parameters in the Uncertainty model. The line graph represents the mean and s.e.m. of the participants’ actual data. The overlap between the shaded area and line graphs show that the Uncertainty model fitted well for each age group.

The Uncertainty model fitted better than these alternative models across age (see Fig. 3a), replicating previous findings in adults^12^. We assessed significance of the model fit difference by using bootstrapping to compute the 95% CI’s of the BIC differences (see Fig. 3b). This showed that the Uncertainty model fitted significantly better than all other models (95% CI of the summed BIC difference between the Uncertainty model and the Sample cost model was 95%CI [− 3086, − 527], the difference with the Threshold model was 95%CI [− 3072, − 636], and with the Count model 95%CI [− 55,095, − 48,774], see Fig. 3b). We performed random effects Bayesian Model Selection for between group comparisons to select the winning model^35^. This returned a high posterior probability of 0.907, reflecting the expected probability that the Uncertainty model generated the data of any randomly selected subject. In other words, there was a high probability of all age groups having the same winning model. The protected exceedance probability showed that the Uncertainty model was most frequent in the comparison set (protected exceedance probability: Uncertainty model = 0.994, Threshold model = 0.005, Sample Cost model = 0.051, Count model = 0.000). In other words, we can be 99,4% confident that the Uncertainty model has a greater posterior probability than any of our other models. Indeed the Uncertainty model predictions matched the data well across age (Fig. 3c; see Supplement for differences between behaviour and each model’s predictions per state). We verified through model recovery that the models were distinguishable and through parameter recovery that the number of trials was sufficient to accurately estimate parameters (see Supplement).

### Prior beliefs, prior belief uncertainty, and uncertainty tolerance underlie age-related differences in trustworthiness information sampling

The winning computational model has four free parameters that were estimated for each participant individually (see “Methods”, “Model fitting and model selection procedure”). Two of these, called $\alpha$_0_ and $\beta$_0_, together make up the individual’s prior belief distribution over *r*, which follows the beta distribution (see “Methods” section “Computational model”). The other two parameters were *k* for uncertainty tolerance, and $\tau$ for decision noise*.* Based on the parameter estimates for $\alpha$_0_ and $\beta$_0_, we calculated two metrics of the participants’ prior belief distributions: prior beliefs and prior belief uncertainty. Since participants do not know the true *r*, they have to marginalize over *r* using their prior distribution to arrive at their prior belief about the probability of reciprocation. This corresponds to the mean of the prior belief distribution (“Methods” Eq. (1)). Moreover, the width of the prior belief distribution reflects the individual’s uncertainty about their prior belief, and is given by the standard deviation of their prior belief distribution (“Methods”, Eq. (2)). In summary, for each subject a prior belief distribution was determined based on free parameters $\alpha$_0_ and $\beta$_0_. The derived metrics of interest are the prior belief and prior belief uncertainty and these were operationalized as the mean and the standard deviation of the prior belief distribution, respectively. Uncertainty tolerance and decision noise were directly estimated from free parameters *k* and $\tau$ (“Methods” Eq. (5)).

**Figure 4:**
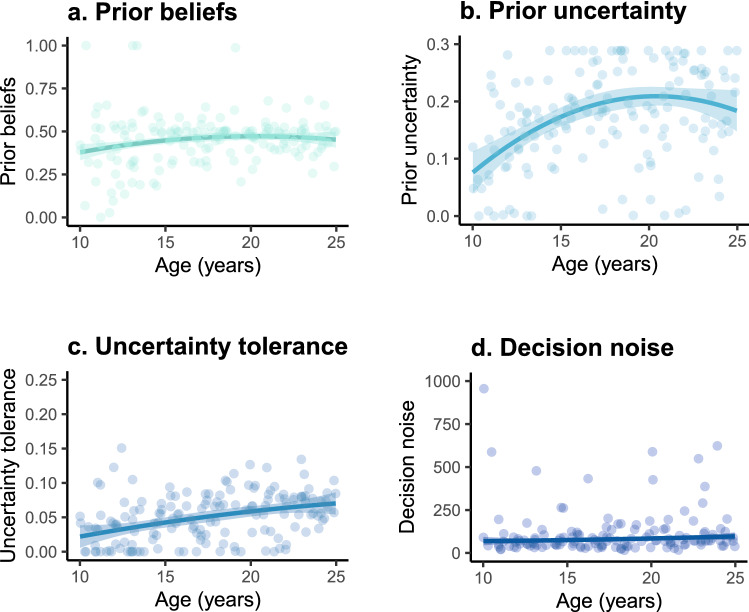
Prior beliefs, prior uncertainty, uncertainty tolerance, and decision noise as a function of age. Each dot represents one individual’s score. Note that a uniform prior corresponds to a prior uncertainty of 0.289, which is the width of the beta distribution for uniform priors. This thereby creates an upper bound for the prior uncertainty. Uncertainty tolerance has the same upper bound as prior uncertainty, as they both reflect the width of the belief distribution. Lower prior uncertainty values reflect a more certain prior. The robust linear regression method with a linear and quadratic age term was used to generate the fit lines.

Given our expected age-differences in prior beliefs, prior belief uncertainty, and uncertainty tolerance, we examined linear and non-linear effects of age on these model-derived metrics using separate robust linear regression models per metric (rlm function; MASS library^36^). We applied a Bonferroni-Holm correction for a total of eight multiple tests, corresponding to four model-derived metrics crossed with two (linear and quadratic) age effects. We found that the *prior beliefs* did not significantly change with age (age linear, *B* = 0.021, *P* = 0.032, *n.s.* after Bonferroni-Holm correction; age quadratic, *B* = − 0.01, *P* = 0.134) (see Fig. 4a). Interestingly, the *prior belief uncertainty* strongly increased from early to mid-adolescence (ages 10–17 years) and then stabilized (Fig. 4b). Results showed a significant linear (*B* = 0.031, *P* < 0.001) and quadratic effect of age (*B* = − 0.020, *P* = 0.002). This suggest an adolescent-emergent increase in uncertainty about their prior beliefs of trustworthiness. Moreover, *Uncertainty tolerance* increased monotonically with age (age linear *B* = 0.014, *P* < 0.001, Fig. 4c; age quadratic *B* = − 0.00, *P* = 0.589), suggesting that adolescents gradually became more uncertainty tolerant with age. Finally, *decision noise* did not significantly change with age (age linear *B* = − 5.69, *P* = 0.574; age quadratic *B* = 20.87, *P* = 0.040, *n.s.* after Bonferroni-Holm correction; Fig. 4d). This shows that the degree to which participants’ sampling choices followed the fitted Uncertainty model predictions did not change with age and therefore gives more confidence in the interpretability of the model derived metrics across adolescence.

### No difference in prior belief distributions between the first and second task-half

To test if the prior beliefs changed during the task, we refitted the Uncertainty model to the first half and second half of the task separately. The prior belief is a model derived metric based on two free parameters in the Uncertainty model, called *α*_0_ and *β*_0_ (see “Methods”). Since different combinations of *α*_0_ and *β*_0_ can result in the same prior belief (but with different uncertainty about those prior beliefs), we conducted these analyses on the *α*_0_ and *β*_0_ parameter estimates rather than on the prior beliefs. Wilcoxon sign-rank tests showed no difference between the first and second task halves in either of the these two parameter estimates (*α*_0_ estimate, *z* = 1.406, *P* = 0.160, median difference < 0.001, 95% CI[− 0.122, 0.315]; *β*_0_ estimate, *z* = 1.133, *P* = 0.257, median difference < 0.001, 95% CI[− 0.478, 0.465]). Moreover, Spearman-rank correlations showed no significant relation between age and the difference between the first and second task halves in either of the two parameter estimates (*α*_0_ estimate *r*_*s*_ = − 0.046, *P* = 0.565; *β*_0_ estimate *r*_*s*_ = − 0.058, *P* = 0.573). Since uncertainty about the prior belief was also a metric derived from the *α*_0_ and *β*_0_ parameters (see “Methods”), this suggests that our findings of the prior beliefs and uncertainty about those beliefs were not likely confounded by changes over trials or age-related changes therein.

## Discussion

Gathering information about outcomes of social interactions to adjust our beliefs about others is critical for successful and adaptive social behaviour. Sampling and using information about others is particularly important during adolescence, as this is a developmental phase in which social cognition and peer relations rapidly develop. We found that adolescents adjusted their sampling quantity to the outcome uncertainty by sampling more when the information was inconsistent. This effect of outcome uncertainty was less strong for early-adolescents compared to older adolescents. Moreover, adolescents trusted (i.e., invested) more often when peers were more trustworthy and this adaptive response to high trustworthiness became stronger with age. These behavioural findings show age differences in social information sampling and in how the information is used in trust decisions. The age differences in information sampling were well captured by a computational model in which a Bayesian belief distribution over trustworthiness is updated with each new sample. The computational model showed that the age differences in sampling could be accounted for by a rapid age-related increase in uncertainty about trustworthiness prior beliefs and a gradual increase in uncertainty tolerance.

The strongest and most striking age difference in the computational model metrics was found in the uncertainty of prior beliefs about trustworthiness. Confirming our hypothesis, we found that from early to mid-adolescence, individuals became more uncertain of their prior beliefs about others’ trustworthiness. This result predicts that with emerging adolescence, individuals should rely less on their prior beliefs and more on the sampled information. This model prediction aligned with the behavioural finding that adolescents increasingly adapted their sampling behaviour to the information inconsistency from early to mid-adolescence. In other words, these findings could be interpreted as early to mid-adolescents becoming more open-minded about possible individual differences in trustworthiness between peers. The increase in uncertainty about prior beliefs is not likely explained by age differences in sampling strategies or task comprehension for at least four reasons. First, we ruled-out alternative computational models that represented alternative sampling strategies, showing that participants of all ages used the same information sampling strategy in their decisions to continue sampling. Second, the instructions were read aloud and in-person by the experimenters who made sure all participants understood the task by asking comprehension questions. Third, age differences in task comprehension cannot account for all age-related effects. Finally, the prior belief distribution parameter estimates did not differ between the first and second task half, which indicates that the age-related increase in prior uncertainty was not due to age differences in sampling strategies or task comprehension as the task progressed.

The increase in uncertainty about prior beliefs may be adaptive given the numerous changes that take place in early to mid-adolescents’ social environment, including changes in social behaviour induced by peers’ pubertal stage and a transition of schools. Interestingly, given that the changes in the adolescent’s environment occur mostly in social contexts but less so in non-social contexts (e.g., physics, such as gravity, mostly remain constant), this further suggests that learning flexibility in adolescents might be especially strong in social contexts. This pertains to learning about others or about the self in relation to a peer group. Future within-subjects studies are required to examine if the age-related increase in uncertainty about prior beliefs is indeed specific to social contexts (such as trusting others), and in which social contexts this may be most pronounced.

We furthermore found that uncertainty tolerance increased linearly with age. This finding at first seems to contradict previous studies on uncertainty tolerance in non-social contexts, which suggest that adolescents are more uncertainty tolerant than adults^28,29,31^. However, in the Uncertainty model, uncertainty updates with each sample and the probability to stop sampling increases once uncertainty drops below the individual’s uncertainty tolerance level. Therefore, uncertainty tolerance should be interpreted in combination with uncertainty about prior beliefs. In our study, mid-adolescents relative to early-adolescents showed increased uncertainty about prior beliefs combined with a smaller increase in uncertainty tolerance. In the model this combination results in an increase in sampling (see model simulations in Fig. 3c). After mid-adolescence, uncertainty about prior beliefs stabilized while uncertainty tolerance continued to increase. In the model, this combination resulted in a decrease in sampling from mid to late-adolescence (Fig. 3c). Although we did not test non-social information sampling in this study, the idea of at least some degree of social specificity is corroborated by previous studies on non-social information sampling. Those studies found that adolescents gathered less information than children or early-adolescents prior to a risky financial decision^31,37^, which diverges from our findings in a trust context. Taken together, our findings of age-related differences in the factors that underlie information sampling fit with our suggestion that mid-adolescents may attempt to learn more about their social environment.

We found no significant age differences in prior beliefs about trustworthiness or in decision noise. Finding no age differences in prior beliefs was somewhat unexpected as some previous studies using repeated trust games show that the first invested amount tends to increase with age, suggesting an age-related increase in initially placed trust^16–19^. However, those findings are inconsistent (see^20,21^). By picking up on subtle, non-significant age differences in prior beliefs, our computational model was able to correctly predict that younger adolescents sampled more relative to older adolescents when the underlying reciprocation probabilities were either high or low (Fig. 3c), again showing that the model predicted sampling behaviour well. Moreover, finding that decision noise did not significantly change with age further strengthens the reliability of the results.

While studies on reinforcement learning during adolescence are scarce, one advantage of this computational modelling approach is using formal model comparisons to assess age-related differences in decision-making strategies^38–40^. For example, a previous study showed that adolescents used different reinforcement learning strategies than adults as the adolescents did not benefit from counterfactual feedback. Such a conlusion would have been challenging to draw without the specific behavioural predictions that resulted from fitting computational models^39^. In the current study, we ruled-out a family of normative models (i.e., Sample Cost model variants), heuristic models that were not based on Bayesian belief distributions (Threshold model variants), and the Count model for insensitivity to gathered evidence. We found that the Uncertainty model showed a good fit and fitted best for all ages, showing that across age, adolescents use their uncertainty in Bayesian belief distributions to update their beliefs about trustworthiness. This is consistent with a previous study on social information sampling costs in adults, where the Uncertainty model also fitted the data best^12^. We show that within this winning model, age-related differences in the parameter estimates accounted well for age differences in sampling behaviour. Our computational modelling and model comparison approach therefore contributes to the field’s understanding of age differences in cognitive strategies of social decision-making.

The sample outcomes in our task were binary (trust/do not trust) decisions. Future studies are needed to identify to which learning situations our age-related findings generalize or diverge. Intuitively, other types of learning situations also allow for variations in uncertainty of a prior belief. Possible domains to explore include continuous reward learning^41,42^ and active learning domains with classification goals^43,44^. The potential applications of our approach extend to understanding how peer-status could influence prior beliefs about others’ behaviour. For instance, children who are frequently socially rejected by their peers may develop different prior beliefs about the trustworthiness of others compared to stably accepted children (e.g., those with experience of frequent rejection may have a highly certain prior belief that others are untrustworthy). Moreover, previous studies used behavioural economic games such as trust games to reveal aberrant social decision-making in psychiatric disorders^45–47^, including anxiety disorders, autism spectrum disorder^48^, borderline personality disorder^47,49,50^, and ADHD^51,52^. In future studies, our task and models might further shed light on how individuals actively sample and use information to initiate or avoid social interactions and how this may depend on aberrant prior beliefs.

## Methods

### Participants and experiment procedure

A total of 157 adolescents (of which 75 boys) completed the experiment (range = 10–24 years, *M* = 17.50, *SD* = 4.34). The sample size was based on prior studies examining age-related differences in uncertainty tolerance within a comparable age-range^27,31^. Participants were screened for colour blindness, psychiatric and neurological disorders, IQ was estimated by using subtests of the WISC and WAIS. IQ scores fell in the normal range (*M* = 107.5, *SD* = 10.9, range = 80–135), and did not correlate with age (*r*_s_ = 0.119, *P* = 0.138), parental social economic status (SES) was estimated by highest educational attainment of the caregiver(s). This sample generally showed a medium to high SES level and SES did not show a relationship with age (Kruskal–Wallis rank sum returned ${\chi }^{2}(4, n=157)$= 6.342, *p* = 0.175; low SES *n* = 8, medium SES *n* = 59, high SES *n* = 90). All procedures were approved by the institutional review board of the Leiden University Medical Center, and performed in accordance with the relevant guidelines and regulations. Written informed consent was given by adult participants, and by their legal guardians in the case of minors (minors provided written assent). This behavioural study was part of a larger imaging study. All participants performed the task in a quiet room near the neuroimaging labs of Leiden University. The task took approximately 30 min to complete (see Supplement for payoff procedure).

### Computational model

*The Uncertainty model* is based on the concept of sampling to reduce uncertainty until the subjective uncertainty tolerance is met. The model consists of four components: a prior belief distribution over the reciprocation probability (*r*), an evolving posterior distribution over *r*, the uncertainty tolerance, and decision noise. As explained above, individuals start with a prior belief distribution when nothing has been sampled yet. This prior belief distribution encompasses both the prior belief and the uncertainty about the prior belief. When information is sampled, the belief distribution is updated and called a posterior distribution. The posterior distribution is therefore a combination of the prior belief distribution and the sampled information. Information is sampled sequentially and each new sample results in a new update. The degree to which the prior belief and the uncertainty about the prior belief updates with each new sample depends on the values of the prior belief, the uncertainty about the prior belief and whether the prior belief is confirmed or disconfirmed by the samples. Examples of how belief updates depend on prior belief distributions and samples are depicted in Fig. 1.

Formally, in our model the *state* is defined by the number of turned green tiles (*n*_+_) and the number of turned red tiles (*n*_−_). The *actions* are to either sample or stop sampling until all 25 tiles are sampled. The true, experimentally set reciprocation probability is denoted as *r*. The model assumes that participants do not know the trustee’s exact trustworthiness when building a Bayesian belief distribution over the possible range of *r*, consistent with the fact that we did not tell our participants what the true *r* was. We assume that the conjugate prior belief distribution over *r* is a beta distribution with parameters $\alpha$_0_ and $\beta$_0_. The decision to trust (invest) can have two outcomes: reciprocation or betrayal. The trust outcome follows a Bernoulli distribution over *r* (i.e., *p*(reciprocation) = *r*). However, since the participant does not know *r* they have to marginalize over their prior distribution over *r,* which is *p*(*r*|*α*_0_*, β*_0_). This marginalization gives the conditional distribution of reciprocation given *α*_0_ and *β*_0_:

1 $$p\left(\mathrm{reciprocation}\right|{\alpha }_{0},{\beta }_{0})= \int p\left(\mathrm{reciprocation}|r\right)p\left(r|{\alpha }_{0},{\beta }_{0}\right)dr= \int rp\left(r|{\alpha }_{0},{\beta }_{0}\right)dr= \frac{{\alpha }_{0} }{{\alpha }_{0}+{\beta }_{0}}$$

This corresponds to the mean of the beta distribution and reflects the prior belief about the reciprocation probability. The width of the prior belief distribution reflects the uncertainty about the reciprocation probability, which is given by the standard deviation of the *prior* belief distribution:

2 $$\begin{array}{c}Uncertainty\left({\alpha }_{0},{\beta }_{0}\right)=\sqrt{\frac{{\alpha }_{0}{\beta }_{0}}{{\left({\alpha }_{0}+{\beta }_{0}\right)}^{2}\left({\alpha }_{0}+{\beta }_{0}+1\right)}}\end{array}$$

When information is sampled, the prior distribution evolves into a posterior distribution over *r* with parameters $\alpha$ and $\beta$. Where $\alpha$= $\alpha$_0_ + *n*_+_ and $\beta$ = $\beta$_0_ + *n*_−_. The posterior distribution is:

3 $$\begin{array}{c}p\left(r|{n}_{+}, {n}_{-}\right)=Beta\left(r;\alpha ,\beta \right)\end{array}$$

The uncertainty of the *posterior* belief is operationalized using the standard deviation of the posterior distribution:

4 $$\begin{array}{c}Uncertainty\left(\alpha ,\beta \right)=\sqrt{\frac{\alpha \beta }{{\left(\alpha +\beta \right)}^{2}\left(\alpha +\beta +1\right)}}\end{array}$$

Sampling decreases uncertainty. At some point, the uncertainty will decrease to the point where it reaches the subject’s uncertainty tolerance *k,* i.e., how much uncertainty is tolerated by the subject. The closer the uncertainty comes to the subject’s uncertainty tolerance level, the smaller the probability that the subject will take another sample becomes. The sampling probability is given through the softmax function, allowing for decision noise $\tau$:

5 $$\begin{array}{c}p\left(\mathrm{sample}|\alpha ,\beta \right)=\frac{1}{{1+e}^{- \frac{Uncertainty\left(\alpha ,\beta \right)-k}{\tau }}}\end{array}$$

*k* reflects more uncertainty tolerance (i.e., an individual with a larger *k* would stop sampling sooner than one with a smaller *k*) and a larger $\tau$ reflects more decision noise.

### Model fitting and model selection procedure

We fitted the models for each participant individually. The models were fitted using Maximum Likelihood Estimation and we used the optimization algorithm as implemented in the fmincon routine in MATLAB (Mathworks) using 100 combinations of starting points to avoid local minima. Four free parameters are fitted for each subject: $\alpha$_0_, $\beta$_0_, *k,* and $\tau$*.* The winning model was selected based on the individually obtained BIC scores, in which the number of free parameters is taken into account to avoid selecting overfitted models. Using bootstrapping, we calculated the 95% CI of the BIC differences between model pairs to assess the significance of this difference (i.e., differences were considered not significant if the confidence interval contained zero). In addition, we used random effects Bayesian Model Selection for between-group comparisons to select the winning model between age groups^35,53^. This method treats models as random effects that could differ between subjects, with an unknown population distribution. The resulting statistics are: 1. the posterior probability, which reflects the probability that a given model generated the data of any randomly selected subject, and 2. the protected exceedance probability, which reflects the probability of one model being more likely than any other model tested.

### Alternative models

We also considered three families of alternative models and found that these did not fit as well as the Uncertainty model (formal descriptions in Supplement): The *Sample Cost model*, which uses the Bayesian belief distribution to compute the normative solution for every state. The *Threshold model* is a heuristic model that does not use Bayesian beliefs distributions. For model comparisons we calculated the difference between model evidence in terms of BIC for each model pair for each subject. The Count model is the simplest heuristic model. In this model, a fixed number of samples are drawn with some variation. Unlike the other models, the decision or stop sampling is therefore not dependent on the outcomes of previous samples. To assess the significance of the model fit differences, we used bootstrapping to compute the 95% confidence intervals of the summed difference in BIC using 10^5^ iterations (for within-model results see Table S1).

## Supplementary Information

Supplementary Information.

## Data Availability

The data of this study are openly available in Open Science Framework https://osf.io/mhr5d/.

## Code availability

The code is available on Github https://github.com/ili-ma/Social_Belief_Updates_Adolescence.
