# Supplementary material for: Uncertainty about others’ trustworthiness increases during adolescence and guides social information sampling
Source: Sci Rep. 2022 May 10;12:7634. doi: 10.1038/s41598-022-09477-2 (PMC9091231; doi:10.1038/s41598-022-09477-2)
Supplement: Supplementary file 1 — Supplementary Information. [file 41598_2022_9477_MOESM1_ESM.docx]

**Uncertainty about others’ trustworthiness increases during adolescence and guides social information sampling**

# ONLINE SUPPLEMENTARY MATERIALS

**Participant recruitment**

Participants were recruited through flyers and presentations at local high schools in the Netherlands, as well as a recruitment website. Based on previous studies (see methods section), we aimed to recruit 160 participants equally divided across 5 age bins (10-12; 13-15; 16-18; 19-21; 22-24 years) with a balanced gender distribution for each age bin. Eventually, 159 people came to the laboratory to participate in our study protocol. One person did not start the study due to anxiety. One person could not finish the sampling experimental task due to time constraints. This led to the reported *N* of 157 adolescents.

**Participant bonus fee**

Participants were instructed upfront that they would be paid based on their performance. More specifically, they were told that the trustees had already decided to reciprocate or not, At the end of the task the computer would select three trustees and the payoff would be based on the sum of those tree trust game rounds. Unbeknownst to the participants, payoff was actually not dependent on trustee decisions, instead three trials were selected and their outcome was averaged to determine payoff. If invested and the generative reciprocation probability was > 0.5 then the outcome was 12 tokens, and 0 tokens if < 0.5. To avoid extreme bonus differences between participants, the payoff trial selection procedure was not fully randomized such that each participant ended with a task performance bonus about between 3 and 9 tokens. The tokens were converted to money as follows: 3 = €1, 4 = €2, 5 = €2, 6 = €3, 7 = €3, 8 = €4, and 9 = €5 and this amount was added to the participation fee.

## **Computational models**

Note that for all models, we tested different variants within each model, and selected the best fitting version for between model comparisons. Here, we denote the best fitting version of each model, for all versions see Ma et al., (2018).

***The Sample Cost model***

*The Sample Cost model* uses the Bayesian belief distribution over trustworthiness to compute the expected utility of sampling and stopping for every possible state in the task through forward reasoning. It consists of four components: prior beliefs over the trustworthiness (*r*), an evolving posterior distribution over *r* iterative maximization of future expected utility under this posterior distribution, and decision noise. The conjugate prior over *r* is a beta distribution with priors $\alpha$_0_ and $\beta$_0_, and parameters $\alpha$= n_+_ + $\alpha$_0_ and $\beta$ = n_-_ + $\beta$_0_, where n_+_ is the number of green samples and n_-_ is the number of red samples. The posterior over *r* is:

$$p\left( r | n_{+}, n_{-} \right)=Beta(r;\alpha,\beta)$$

Investing results in either reciprocation (outcome = 1 with probability *r*, then the investment amount is multiplied by m = 2) or betrayal (outcome = 0 with probability 1 - *r*, then the investment amount is multiplied by m = 0). The agent does not know *r* and therefore has to marginalize over *r*, using the current posterior. This gives the conditional distribution of outcome given $\alpha$ and $\beta$:

$p\left( \mathrm{outcome}=1 \right|\alpha,\beta)= \int p\left( \mathrm{outcome}=1 | r \right)p\left( r | \alpha,\beta\right)dr=\int rp\left( r | \alpha,\beta\right)dr=\frac{\alpha}{\alpha+\beta}$ (1)

The expected utility of *not* investing is U_0_ = 1. The expected utility of investing, U_1_, with a free parameter $\lambda$for risk attitude becomes:

$$\begin{aligned} U_{1}\left( \alpha,\beta\right) =E\left[ \mathrm{outcome} | \alpha,\beta\right]- \lambda\mathrm{Var}\left[ \mathrm{outcome} | \alpha,\beta\right] \\ = \frac{m\alpha}{\alpha+\beta} - \frac{\lambda m^{2}\alpha\beta}{\left( \alpha+\beta\right)^{2}} \\ \#\left( 2 \right) \end{aligned}$$

The agent can decide between sampling (a = 1) or stopping (a = 0) at any time except when t = T+1, then all boxes are opened and only stopping (a = 0) is possible. The value of a state-action pair is given by the Bellman equations (Bellman, 1952). Specifically, the expected value of the state ($\alpha, \beta$) is the higher of the expected utilities of not investing and investing:

$$\begin{aligned} Q_{t}\left( \alpha,\beta;a=0 \right)=\max\left\{ U_{0},U_{1}\left( \alpha,\beta\right) \right\}\#\left( 3 \right) \end{aligned}$$

When t = T+1, the value of the state ($\alpha+\beta$) is V_t_($\alpha, \beta$) = Q_T+1_($\alpha, \beta$; a = 0). At earlier times, V_t_ is the larger of the expected utilities:

$$\begin{aligned} V_{t}\left( \alpha,\beta\right)=\max\left\{ Q_{t}\left( \alpha,\beta;a=0 \right),Q_{t}\left( \alpha,\beta;a=1 \right) \right\}\#\left( 4 \right) \end{aligned}$$

The expected value of a sampling action (a = 1) at time *t* in the state $\alpha, \beta$ subtracting the subjective cost of a sample *c* is:

$$\begin{aligned} Q_{t}\left( \alpha, \beta; a=1 \right)=\frac{\alpha V_{t+1}\left( \alpha+1,\beta\right)+\beta V_{t+1}\left( \alpha, \beta+1 \right)}{\alpha+ \beta}-c\#\left( 5 \right) \end{aligned}$$

By starting with the final state (when *n* = T), we can obtain the Sample Cost solution for every possible state (dynamic programming). In the Sample Cost model, the decision variable (DV), is the difference between the utilities of sampling and stopping:

$$\begin{aligned} DV\left( \alpha,\beta\right)=Q_{t}\left( \alpha,\beta;a=1 \right)-Q_{t}\left( \alpha,\beta;a=0 \right)\#\left( 6 \right) \end{aligned}$$

The Sample Cost policy would be to sample when the DV is positive; however, we introduce decision noise through a softmax function:

$$p\left( \mathrm{sample} | \alpha,\beta\right)=\frac{1}{1+e^{-\frac{DV\left( \alpha,\beta\right)-k}{\tau}}}$$

This model version has six free parameters: $\alpha_{0},\beta_{0}, c, \lambda, \tau, \mathrm{and} k$ which were fitted for each subject.

***The Threshold model***

In the *Threshold Model,* the agent keeps track of the absolute difference between positive and negative information and stops sampling when this difference reaches a bound. However, to be consistent with our other models and with most of the value-based decision literature, we use soft rather than hard bounds. The bound *b* takes three possible values, depending on the sign of n_+_ - n_-_ :

$$\begin{aligned} b=\left\{ \begin{matrix} b_{+} & \mathrm{if}n_{+}>n_{-} \\ \frac{b_{+}+b_{-}}{2} & \mathrm{if}n_{+}=n_{-} \\ b_{-} & \mathrm{if}n_{+}< n_{-} \end{matrix} \right.\#\left( 7 \right) \end{aligned}$$

The probability that the agent stops sampling is a logistic function of the difference between this decision variable and a bound *b*:

$$\begin{aligned} p\left( \mathrm{sample} | n_{+}, n_{-} \right)=\frac{1}{1+e^{-\frac{DV\left( n_{+}, n_{-} \right)-b}{\tau}}}\#\left( 8 \right) \end{aligned}$$

This model version has three free parameters: *b*_+_, *b*_-_$, \mathrm{and}\tau$ which were fitted for each subject.

***The Count model***

In the count model, the agent takes a fixed number of samples *k* with some variation. This model represents a simple heuristic where the decision to sample another tile is not determined by the outcomes of the previous samples. We added this model to test if some individuals and especially younger participants might have a strategy of for example always sampling five tiles before making their decision to trust or not trust. The softmax function allows for decision noise:

$$\begin{aligned} p\left( \mathrm{sample} | n_{+}, n_{-} \right)=\frac{1}{1+e^{\frac{{(n}_{+}+ n_{-})-k}{\tau}}} \#\left( 9 \right) \end{aligned}$$

## This model has two free parameters: *k* and $\tau$ which were fitted for every subject.

## **Within-model comparisons results**

For each model, we first fitted the basic version - the version with the fewest free parameters - and then tested whether adding a free parameter significantly improved the model fit (also see (Ma et al., 2018) for more details). The assessment of these additional free parameters was based on the trust game literature. Here, we describe the additionally tested free parameters for each model across all participants. The *Count model* is not included in the within-model comparisons, as it only had one version of the model, which had two free parameters; one for the fixed number of samples and one for variability.

*Uncertainty model: prior beliefs improve the model fit.* For the same reasons as described earlier, we tested the improvement in model fit when adding a prior belief, also estimated for each subject. In the basic version of our model, we used an uninformative, uniform prior (i.e., *α*_0_ = 1 and *β*_0_ = 1). In the model version with prior beliefs, we fitted *α*_0_ and *β*_0_ as free parameters for every subject. We found that allowing for a subjective prior improved the model fit (Table S1.0). The median of individual prior means was *r* = 0.47 (bootstrapped 95% CI [0.45, 0. 50]).

*Sample Cost model: prior beliefs and risk attitude both improve the model fit.* The repeated trust game literature suggests that subjective prior beliefs (Chang et al., 2010) and betrayal aversion (Aimone & Houser, 2012) both play important roles in determining individual differences in trust. In our paradigm, an individual’s prior belief about trustworthiness is a beta distribution with two free parameters (*α*_0_ and *β*_0_). Betrayal attitude is operationalized as the variance of the outcome, multiplied by a free parameter *λ*, which is subtracted from the expected utility of trusting. The median betrayal attitude parameter was estimated at 0.16 (bootstrapped 95% CI [0.10, 0.23]), which shows that participants were overall betrayal-averse.

*Threshold model: asymmetric bounds but not collapsing bounds improved the model fit.* The literature on Drift Diffusion Models in perception literature suggests that the model fits better with collapsing bounds that reflect an urgency signal (Tajima et al., 2016), or asymmetric bounds (Mulder et al., 2012) as positive sample outcomes might be weighted differently than negative outcomes. Using separate bounds for positive than for negative samples did indeed improve the model fit. Second, we tested the model when the bounds “collapse” to zero over time. This did not improve the model fit from the model with asymmetric bounds.

Table S1.0. Within-model comparison results

|  |  | 95% CI | |
| --- | --- | --- | --- |
|  | Summed $\Delta$BIC | Lower bound | Upper bound |
| Uncertainty basic vs. priors | 2013 | 925 | 3316 |
| Sample Cost basic vs. risk attitude | 1597 | 1006 | 2293 |
| Sample Cost risk attitude vs. risk attitude + priors | 791 | 398 | 1205 |
| Threshold basic vs. two bounds | 44987 | 38479 | 51847 |
| Threshold collapsing bound vs two bounds | 847 | 501 | 1198 |

95% CI = Bootstrapped 95% confidence interval of the summed difference between model fits. Smaller BIC values indicate better fit. Thus, positive values indicate a better fit for the second model. The models were fitted to the data at the individual level using a log likelihood optimization algorithm as implemented in the fmincon routine in MATLAB (©Mathworks). The optimization was iterated 100 times with varying initiations to avoid local minima. Because of the summation of the difference, large positive or negative numbers therefore reflect that one model wins consistently, i.e. for most subjects.

**Between- model comparison results**

We then compared the best fitting version of each model with the best fitting model of the other models. Table S1.1 shows the results which indicate that the Uncertainty model fitted best.

Table S1.1. Between model comparisons

| **Pairwise comparison between models** | **Summed** $\boldsymbol{\Delta}$**BIC and 95% CI** | **Winning model** | **Correlation between age and** $\boldsymbol{\Delta}$**BIC** |
| --- | --- | --- | --- |
| Uncertainty - Sample Cost | -1685 [-3086, -527] | **Uncertainty** | *r_s_* = -0.059,  *P =* 0.464 |
| Uncertainty - Threshold | -1741 [-3072, -636] | **Uncertainty** | *r_s_* = 0.101,  *P =* 0.221 |
| Uncertainty - Count | -10228 [-10919, -9542] | **Uncertainty** | *r_s_* = 0.076,  *P =* 0.346 |
| Sample Cost - Threshold | -56 [-627, 504] | **None** | *r_s_* = 0.031,  *P =* 0.711 |
| Sample Cost - Count | -8531[-9911, -7022] | **Sample Cost** | *r_s_* = 0.058,  *P*  =0.474 |
| Threshold - Count | -8439[-9779, -6979] | **Threshold** | *r_s_* = 0.101,  *P* = 0.209 |

Lower BIC values indicate a better fit, thus showing that the Uncertainty model fits significantly better than all other models. BIC scores were computed for each participant and each model. The BIC scores of a model pair (left column) was then subtracted from each other, thereby obtaining one difference score per participant for each model pair. The middle column shows the sum of the difference across participants and the 95% confidence interval of the BIC difference, computed using bootstrapping with 10^5^ iterations. The last column shows the Spearman-rank correlation between age and each model pair’s BIC difference.

**Number of samples as a function of outcome uncertainty and age**

For the analyses of the number of samples, we first calculated the outcome uncertainty, which is the variance in the Bernoulli distribution, where *r* is the probability of reciprocation:

Outcome uncertainty = *r*(1- *r*)

A robust linear mixed model (package ‘robustlmm’(Koller, 2016)) was run to examine effect of outcome uncertainty, age (linear, quadratic) and their interactions on sampling behaviour. Subject was included as a random intercept to account for the within-subject nature of the data. For transparency we report the full mixed effects model in R code here:

SamplingModel <- rlmer(number of samples ~ scale(outcome uncertainty) * scale(age linear) + scale(age quadratic) + (1|subject), data = df)

where the linear and quadratic age terms were defined using the poly() function in R. This returned the results in Table S2.0:

Table S2.0. Results mixed effects model number of samples as function of age and outcome uncertainty

| Predictors | $B$ | 95% CI | *p*-value |
| --- | --- | --- | --- |
| Intercept | 16.64 | 15.70 – 17.58 | **<0.001** |
| Outcome uncertainty | 2.13 | 2.04 – 2.23 | **<0.001** |
| Age linear | -0.88 | -1.82 – 0.06 | 0.065 |
| Age quadratic  Outcome uncertainty x Age linear  Outcome uncertainty x Age quadratic | -0.24  0.36  -0.14 | -1.18 – 0.7  0.27 – 0.45  -0.23 - -0.05 | **0.617**  **<0.001**  **0.003** |
| **Random Effects** | | | |
| σ^2^ | 19.85 | | |
| τ_00_ _subject_ | 33.96 | | |
| ICC | 0.63 | | |
| N _subject_ | 157 | | |
| Observations | 9420 | | |
| Marginal R^2^ / Conditional R^2^ | 0.093 / 0.666 | | |

**Post hoc test for the number of samples as a function of outcome uncertainty per age group**

To further examine the interaction effect, we created age bins (consistent with Figure 2c in the main manuscript) and compared the slopes for outcome uncertainty between the age groups using the emtrends() function (package ‘emmeans’(Lenth et al., 2021)). This showed that early adolescents differed significantly from all other age groups (Table S2.1).

Table S2.1. Results post-hoc pairwise comparison per age group

| Contrast age groups | *B* | *p*-value |
| --- | --- | --- |
| 1-2 | -11.65 | **<0.0001*** |
| 1-3 | -12.4 | **<0.0001*** |
| 1-4 | -8.97 | **<0.0001*** |
| 1-5 | -14.46 | **<0.0001*** |
| 2-3 | -0.75 | 0.987 |
| 2-4 | 2.68 | 0.358 |
| 2-5 | -2.81 | 0.316 |
| 3-4 | 3.43 | 0.1386 |
| 3-5 | -2.06 | 0.638 |
| 4-5 | -5.49 | **0.002*** |

Note: Note: reported p-values are Tukey corrected. *p-values are significant at the Tukey corrected level. Age group 1 are 10-12 year olds, age group 2 are 13-15 year olds, age group 3 are 16-18 year olds, age group 4 are 19-20 year olds, and age group 5 are 21-24 year olds.

**Number of samples as a function of reciprocation probability and age**

We also created bins for the experimentally set probability of reciprocation (consistent with Figure 2c in the main manuscript). A similar robust linear mixed model was run to examine effect of reciprocation probability, age (linear, quadratic) and their interactions on sampling behaviour. Subject was included as a random intercept to account for the within-subject nature of the data. The results can be found in Table S2.2.

Table S2.2. Results number of samples mixed model on reciprocation probability

| Predictors | $B$ | 95% CI | *p*-value |
| --- | --- | --- | --- |
| Intercept | 16.57 | 15.62-17.52 | **<0.001*** |
| Reciprocation probability | 0.72 | 0.62-0.83 | **<0.001*** |
| Age linear | -0.9 | -1.85-0.05 | 0.063 |
| Age quadratic  Reciprocation probability x Age linear  Reciprocation probability x Age quadratic | -0.26  -0.17  -0.07 | -1.21 – 0.7  -0.28 – 0.07  -0.04 - 0.17 | **0.598**  **<0.001***  **0.216** |
| **Random Effects** | | | |
| σ^2^ | 25.69 | | |
| τ_00_ _subject_ | 34.7 | | |
| ICC | 0.57 | | |
| N _subject_ | 157 | | |
| Observations | 9420 | | |
| Marginal R^2^ / Conditional R^2^ | 0.023 / 0.584 | | |

**Post-hoc test for number of samples as a function of age per reciprocation probability**

We next used the emtrends() function from the emmeans package to compare the linear slopes of the age effect between the different reciprocation probabilities. The effect of age on the number of samples was strongest in the highest and lowest reciprocation probabilities (Table S2.3).

Table S2.3. Post-hoc age effect on number of samples per reciprocation probability bin

| Reciprocation probability | $B$ | *p*-value |
| --- | --- | --- |
| 0.0 | -1.051 | **0.0312*** |
| 0.2 | -0.794 | 0.1035 |
| 0.4 | -0.354 | 0.4683 |
| 0.6  0.8  1.0 | -0.361  -1.130  -1.564 | 0.4594  **0.0205***  **0.0014*** |

Note: reported p-values are Tukey corrected. *p-values are significant at the Tukey corrected level.

**Invest decisions (trust / do not trust) as a function of reciprocation probability and age**

A generalized linear mixed effects model (package ‘lme4’(Bates et al., 2015)) was run to examine effect of reciprocation probability, age (linear, quadratic) and their interactions on the decision to invest (trust) or not. Subject was included as a random intercept to account for the within-subject nature of the data. For transparency we report the full mixed effects model in R code here:

InvestModel <- glmer(Invest decision ~ scale(reciprocation probability) * (scale(age linear) + scale(age quadratic)) + (1| subject), data = investdata, family = binomial, control = glmerControl(optCtrl = list(maxfun = 1e+9), optimizer = c("bobyqa")))

where the linear and quadratic age terms were defined using the poly() function in R. This returned the results in Table S3.0

Table S3.0. Results invest decisions mixed model

| Predictors | Odds Ratios | 95% CI | *p*-value |  |
| --- | --- | --- | --- | --- |
| Intercept | 0.62 | 0.53 – 0.72 | **<0.001** |  |
| Reciprocation probability | 30.04 | 25.92 – 34.81 | **<0.001** |  |
| Age linear | 1.00 | 0.86 – 1.16 | **0.987** |  |
| Age quadratic | 1.04 | 0.90 – 1.21 | **0.592** |  |
| Reciprocation probability x age linear  Reciprocation probability x age quadratic | 2.06  0.76 | 1.80 – 2.36  0.67 – 0.86 | **<0.001**  **<0.001** |  |
| **Random Effects** | | | | |
| σ^2^ | 3.29 | | | |
| τ_00_ _subject_ | 0.70 | | | |
| ICC _subject_ | 0.18 | | | |
| N _subject_ | 157 | | | |
| Observations | 9420 | | | |
| Marginal R^2^ / Conditional R^2^ | 0.753 / 0.797 | | | |

**Post-hoc test for invest decisions**

To further examine the interaction effects between age and reciprocation probability on invest decisions, we created reciprocation probability bins and compared the slopes for age using the emtrends() function (package ‘emmeans’ (Lenth et al., 2021)). This showed that the age differences were most pronounced in the lowest and highest reciprocation probabilities (Table S3.1).

Table S3.1. post hoc test for the effect of age on invest decisions per reciprocation probability

| Reciprocation probability | Odds ratios | 95%CI | *p*-value |
| --- | --- | --- | --- |
| 0.0 | 0.37 | 0.18-0.72 | **0.004*** |
| 0.2 | 0.63 | 0.39-1.04 | 0.071 |
| 0.4 | 0.91 | 0.74-1.11 | 0.352 |
| 0.6 | 1.10 | 0.90-1.34 | 0.348 |
| 0.8 | 1.93 | 1.46-2.56 | **<0.001*** |
| 1.0 | 2.88 | 1.89-4.40 | **<0.001*** |

Note: Note: reported p-values are Tukey corrected. *p-values are significant at the Tukey corrected level. *p-values are significant at the Tukey corrected level.

## **Expected reward increased with age**

Additionally, we examined if the expected values based on the trust decisions varied with age. We first normalized the outcomes on the endowment of 6 tokens. Thus, the expected value for not investing is 1. The expected value for investing is computed as the multiplier of 2 times the reciprocation probability. Since the reciprocation probabilities were 0.0, 0.2, 0.4, 0.6, 0.8, and 1.0, the possible average expected values ranged from $\frac{0 + 0.4 + 0.8 + 1 + 1 + 1}{6}=0.67$ to $\frac{1+1+1+1.2+1.6+2}{6}=1.3$. All subjects (with exception of one), had an average expected reward higher than 1 (see Figure S1). This confirms that all subjects would - on average - have gained money on top of their original endowment by trusting when trusting was beneficial. However, younger adolescents earned less on this task than older adolescents, consistent with their deviations in low (0.0) and high (0.8, 1.0) investment probabilities. This was shown by a significant correlation between age and the average expected reward in the task (*r_s_* = 0.387, *P <* 0.001).

**Figure S1**. Expected reward per subject as function of age. The expected reward increases with age. Errorbars indicate the s.e.m. The line and shaded region indicates the mean and s.e.m across subjects. Color indicates age in months

**Differences between human data and fitted model predictions per state**

a. Difference between behavior and model predictions per state


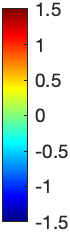

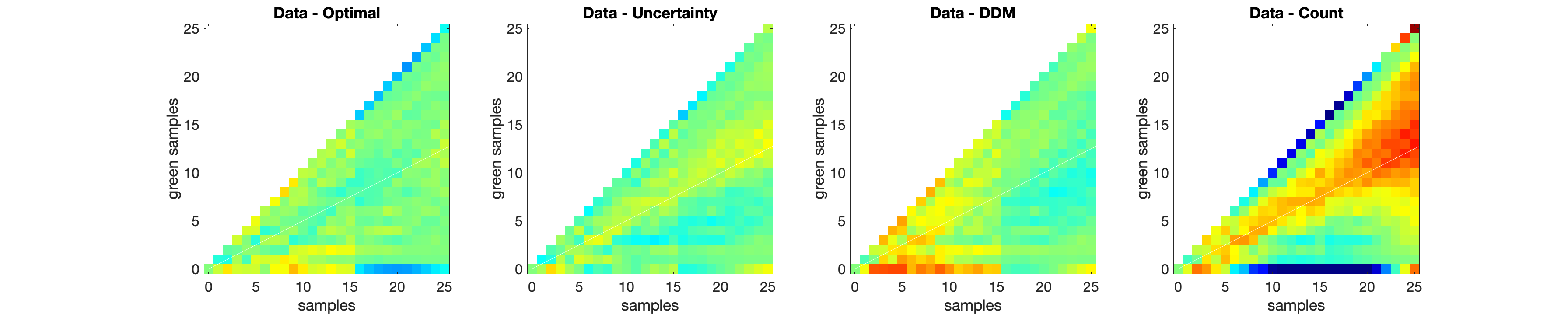


b. Observations per state in human behavior

**Figure S2.** Model predictions and data per state. **A.** Heat plots of the difference between the human data and the model predictions on each observed state. Warmer colors indicate more samples in the human data than in the model predictions. Colder colors indicate more samples predicted by the model than observed in human data. The x-axis shows the number of samples, the y-axis shows the number of samples that were green tiles. As such the triangle shape covers the entire state space (e.g., the bottom right corner is the situation where 25 tiles were turned and none were green, and the top right corner is the scenario where 25 tiles were turned and all were green). The figure was generated for each subject, then averaged over subjects. **B.** Heat plot of how often humans reached each observed state. This plot is also averaged over subjects. Warmer colors indicate more frequently encountered states. Frequently encountered states are more important than rarely occurring states when evaluating the differences between the model predictions and data.

## **Model recovery**

We performed model recovery and verified that the models were distinguishable. To this end, we simulated data with each model and subsequently fitted the simulated data to each model. If the model is recoverable, then the best fitting model should be the model that generated the data. We show that this was indeed the case as the model fit was always best for the model that generated the data (Table S4.0).

Table S4.0. Model recovery results

|  |  | 95% CI | |  |
| --- | --- | --- | --- | --- |
|  | Summed $\Delta$BIC | Lower bound | Upper bound | |
| **Data generated by the Uncertainty model** |  |  |  | |
| Uncertainty vs. Sample Cost | -849 | -1057 | -654 | |
| Uncertainty vs. Threshold | -568 | -861 | -293 | |
| Uncertainty vs Count | -7014 | -8129 | -5859 | |
| **Data generated by the Sample Cost model** |  |  |  | |
| Sample Cost vs. Uncertainty | -795 | -313 | -541 | |
| Sample Cost vs. Threshold | -537 | -908 | -249 | |
| Sample Cost vs Count | -7350 | -8675 | -5978 | |
| **Data generated by the Threshold model** |  |  |  | |
| Threshold vs. Sample Cost | -827 | -964 | -689 | |
| Threshold vs. Uncertainty | -654 | -894 | -448 | |
| Threshold vs Count | -6102 | -6923 | -5179 | |
| **Data generated by the Count model** |  |  |  | |
| Count vs. Sample Cost | -2193600 | -2195896 | -2191370 | |
| Count vs. Uncertainty | -2563 | -2917 | -2224 | |
| Count vs. Threshold | -2190400 | -2192665 | -2188118 | |

95% CI = Bootstrapped 95% confidence interval of the summed difference between model fits. Negative values indicate a better fit of the model that generated the data. The data were generated using the participants’ parameter estimates and shows that all models were recoverable.

To further clarify the model recovery results, we also assessed the model identifiability in terms of the percentage of correctly recovered models. Table S4.1 below shows how often the model that was used to simulate the data was indeed the winning model.

Table S4.1. Model recovery results in percentages

| Model used for recovery | | | | | |
| --- | --- | --- | --- | --- | --- |
|  |  | Uncertainty model | Sample Cost model | Threshold model | Count model |
|  | Uncertainty model | 70% | 0% | 25% | 5% |
|  | Sample Cost model | 0% | 80% | 15% | 5% |
|  | Threshold model | 0% | 0% | 95% | 5% |
|  | Count model | 0% | 0% | 0% | 100% |

Cells show how often the model used for recovery won. The rows in the table show which model generated the data. The columns show the model used for recovery. We randomly selected the estimates of 20 subjects to simulate data with an equal number of trials as in the actual task. The cells show how often the model that generated the data was the winning model, expressed in percentages.

Model used for data simulation

## **Parameter recovery**

To check if the parameters were recoverable, we simulated data with each model using varying parameter values as inputs. We randomly selected the estimates of all subjects to simulate data with an equal number of trials as in the actual task. We then fitted that simulated data to the model to check if the parameters values that generated the data were correctly estimated. We subtracted the estimated parameters of the simulation from the actual input parameters and calculated the bootstrapped 95% confidence interval and found no significant difference between the input parameters and the estimated parameter values. This returned: Uncertainty tolerance median difference = -0.0001, 95% CI [-0.000, 0.000]; *α*_0_ median difference = -0.386, 95% CI [-0.590, 0.000]; *β*_0_ median difference = -0.250, 95% CI [-0.580, 0.000]). This suggests that the parameters were indeed recoverable. Furthermore, we plotted the simulated parameter values against the recovered estimates and found that the parameters of all models recovered reasonably well, see figures S3.1 for the Uncertainty model, S3.2 for the Sample Cost model, S3.3 for the Threshold model, and S3.4 for the Count model.

**Figure S3.1**. Uncertainty model parameter recovery results. X-axis shows the simulated parameter values or model metric. Y-axis shows the recovered parameters or model metric. Prior uncertain is the model metric ‘prior uncertainty’. Uncertainty tol = uncertainty tolerance. The diagonal represents perfect recovery. The decision noise data points of 29 subjects are not shown in the decision noise graph, as they were much higher, which made the variability at the lower end difficult to see. However, all data points are shown in the other three graphs, including those of the 29 subjects with high decision noise.

**Figure S3.2.** Sample Cost model parameter recovery results. X-axis shows the simulated parameter values or relevant model metric. Y-axis shows the recovered parameters or relevant model metric. The diagonal represents perfect recovery. The decision noise data points of 17 subjects are not shown in the decision noise graph, as they were much higher, which made the variability at the lower end difficult to see. However, all data points are shown in the other three graphs, including those of the 17 subjects with high decision noise.

**Figure S3.3.** Parameter recovery for the Threshold model. X-axis shows the simulated parameter values or model metric. Y-axis shows the recovered parameters or model metric. The diagonal represents perfect recovery.

**FigureS3.4.** Parameter recovery for the Count model. X-axis shows the simulated parameter values or model metric. Y-axis shows the recovered parameters or model metric. The diagonal represents perfect recovery.

**References**

Aimone, J. A., & Houser, D. (2012). What you don’t know won’t hurt you: A laboratory analysis of betrayal aversion. *Experimental Economics*, *15*(4), 571–588.

Bates, D., Mächler, M., Bolker, B., & Walker, S. (2015). *Fitting linear mixed-effects models using lme4*. *67*, 1–48.

Bellman, R. (1952). On the theory of dynamic programming. *Proceedings of the National Academy of Sciences of the United States of America*, *38*(8), 716.

Chang, L. J., Doll, B. B., van’t Wout, M., Frank, M. J., & Sanfey, A. G. (2010). Seeing is believing: Trustworthiness as a dynamic belief. *Cognitive Psychology*, *61*(2), 87–105.

Koller, M. (2016). robustlmm: An R package for robust estimation of linear mixed-effects models. *Journal of Statistical Software*, *75*(1), 1–24.

Lenth, R., Buerkner, P., Herve, M., Love, J., Riebl, H., & Singmann, H. (2021). Emmeans: Estimated marginal means, aka least-squares means (1.6.3). *R Package*.

Ma, I., Sanfey, A. G., & Ma, W. J. (2018). The Cost of Appearing Suspicious? Information Gathering Costs in Trust Decisions. *BioRxiv*, 495697.

mulder, martijn, Wagenmakers, E.-J., Ratcliff, R., & Forstmann, B. (2012). Bias in the brain: A diffusion model analysis of prior probability and potential payoff. *Journal of Neuroscience*, *7*. https://doi.org/10.1523/JNEUROSCI.4156-11.2012

Tajima, S., Drugowitsch, J., & Pouget, A. (2016). Optimal policy for value-based decision-making. *Nature Communications*, *7*, 12400.
